# What Is the Impact of Antibiotic Resistance Determinants on the Bacterial Death Rate?

**DOI:** 10.3390/antibiotics14020201

**Published:** 2025-02-14

**Authors:** Bruno T. S. Luz, João S. Rebelo, Francisca Monteiro, Francisco Dionisio

**Affiliations:** cE3c—Centre for Ecology, Evolution and Environmental Changes & CHANGE, Global Change and Sustainability Institute, Faculdade de Ciências, Universidade de Lisboa, 1749-016 Lisboa, Portugal; fc54303@alunos.fc.ul.pt (B.T.S.L.); fc45634@alunos.fc.ul.pt (J.S.R.)

**Keywords:** bacterial death, bacterial persistence, antibiotic resistance, epistasis, bacterial fitness, conjugative plasmid, nalidixic acid, rifampicin

## Abstract

**Objectives:** Antibiotic-resistant bacteria are widespread, with resistance arising from chromosomal mutations and resistance genes located in the chromosome or in mobile genetic elements. While resistance determinants often reduce bacterial growth rates, their influence on bacterial death under bactericidal antibiotics remains poorly understood. When bacteria are exposed to bactericidal antibiotics to which they are susceptible, they typically undergo a two-phase decline: a fast initial exponentially decaying phase, followed by a persistent slow-decaying phase. This study examined how resistance determinants affect death rates during both phases. **Methods:** We analyzed the death rates of ampicillin-exposed *Escherichia coli* populations of strains sensitive to ampicillin but resistant to nalidixic acid, rifampicin, or both, and bacteria carrying the conjugative plasmids RN3 or R702. **Results:** Single mutants resistant to nalidixic acid or rifampicin decayed faster than sensitive cells during the early phase, whereas the double-resistant mutant exhibited prolonged survival. These contrasting impacts suggest epistatic interactions between both chromosomal mutations. Persistent-phase death rates for chromosomal mutants did not differ significantly from wild-type cells. In contrast, plasmid-carrying bacteria displayed distinct dynamics: R702 plasmid-bearing cells showed higher persistent-phase death rates than plasmid-free cells, while RN3 plasmid-bearing cells exhibited lower rates. **Conclusions:** Bactericidal antibiotics may kill bacteria resistant to other antibiotics more effectively than wild-type cells. Moreover, epistasis may occur when different resistance determinants occur in the same cell, impacting the bactericidal potential of the antibiotic of choice. These results have significant implications for optimizing bacterial eradication protocols in clinical settings, as well as in animal health and industrial food safety management.

## 1. Introduction

Growth rate is a critical determinant of bacterial fitness, as are the lag and death phases. At first, one might assume that shorter lag phases benefit bacteria. However, longer lag phases can be advantageous for bacteria to tolerate antibiotics [1]. Fridman et al. studied how bacterial populations adapt to intermittent exposure to antibiotics. Interestingly, they showed that the initial response to antibiotic stress was tolerance, achieved by adjusting single-cell lag times to match the duration of antibiotic exposure intervals [1]. Here, natural selection acted non-trivially: surviving cells took longer to reach the exponential phase, having longer lag times. Death is also an important component of bacterial fitness. If a toxin is present in the medium, cells that take longer to die may have an advantage if the toxin degrades or is flushed out over time [2,3]. Since natural selection does not occur among dead cells, metabolism and/or cell replication of living cells should determine death speed.

How fast do bacteria die when exposed to bactericidal toxins or antibiotics to which they are sensitive? After a few tens of minutes, bacterial populations decay exponentially until more than 99% of the cells die. The resilient cell population that remains alive are the persistent cells, which consist of slow-growing/dormant bacteria with lower metabolic activity, tolerant to antibiotics. Persister cells can exit from this dormant state and resume growth after antibiotic removal, acquiring a similar antibiotic-sensitive behavior to the initial cell culture [4,5,6,7,8,9].

Although occurring spontaneously in bacterial populations, persistence can be induced by environmental conditions, such as exposure to antibiotics [10]. For example, (p)ppGpp synthetase Rsh promotes persister cell formation in *B. abortus* stationary phase treated with rifampicin and enrofloxacin [11,12], but not in the *Staphylococcus aureus* stationary phase treated with ciprofloxacin or gentamicin. Moreover, resistance to an antibiotic can promote persistence. Braetz and colleagues demonstrated that *Salmonella enterica* serovar Typhimurium, which are less susceptible to ciprofloxacin compared to wild-type cells, exhibited higher persistence levels than their wild-type counterparts [13]. Since most clinically relevant bacteria resist at least one antibiotic [14,15,16], an important question emerges: if a specific antibiotic is used to eliminate bacterial cells, what is the impact of resistance determinants on persistence levels and, consequently, death rates?

The mechanisms underlying the death of persister populations remain a topic of debate. If a genetic program governs persistence, persister populations should decay following first-order kinetics, characterized by a single rate constant. In this scenario, these populations would decay exponentially, corresponding to the stochastic breakdown of the genetic mechanism responsible for persistence [17]. An alternative to this hypothesis is that persistence would be the result of different kinds of glitches and errors in cell division [18]. Hence, this would generate a heterogeneous population composed of several bacterial subpopulations with their own exponential rates [17]. Consequently, persister populations would decay proportionally to a power law *t^β^* (where t represents time, and *β* < 0) and not proportionally to an exponential, *e^k.t^* (with k < 0) [19,20]. The same work further shows that *β* should have a specific value, *β* = −2; therefore, persister populations should decay according to *t*^−2^ [17]. With different reasoning based on network modeling [21], Kaplan et al. have also shown that if persistence is a state caused by the disruption of biomolecular networks, persister populations should decay according to a power law [22].

Meanwhile, we have argued that because the heterogeneous subpopulation rejuvenates later than the non-persister cells, their rejuvenation constant should be lower than that of the non-persister cells, k_1_ [23]. Therefore, the population should decay according to $\frac{1}{t^{2+\delta }}$ (instead of $\frac{1}{t^{2}}$), where *δ* = −*β* − 2 is a positive number close to zero and β is close but slightly lower than −2, as experimentally observed [17,23]. We have also shown that if the persister population stops replicating due to errors in cell division, there is a relationship between the rejuvenation constant of the non-persister population *k*_1_ and *β*: $1-\left(1-k_{1}t\right)e^{k_{1}t}=t^{2+\beta }$. To our knowledge, this relationship remains untested. One possible way to test this relationship is to study different strains with different decay rates *k*_1_.

This study aimed to investigate whether the presence of resistance determinants affects persistence levels or alters the death rate of the persister population. Specifically, we examined how two chromosomal mutations conferring antibiotic resistance and two naturally occurring plasmids influence bacterial persistence levels and decay dynamics. During the early phase, single mutants resistant to nalidixic acid or rifampicin decayed more rapidly than antibiotic-sensitive cells, while the double-resistant mutant demonstrated improved survival. These results suggest epistatic interactions between these chromosomal resistance mutations [24]. In the persistent phase, however, the death rates of these mutants were comparable to those of wild-type cells. Plasmid-bearing bacteria also exhibited interesting behavior. Cells harboring the R702 plasmid showed increased death rates during the persistent phase relative to plasmid-free cells, whereas those carrying the RN3 plasmid displayed reduced death rates. These differences were independent of growth rates. We demonstrated that both exponential and power law mathematical functions accurately describe the decay of persistence populations. Moreover, when assuming that the persister populations decay according to power laws, we were able to verify some predictions of the mathematical relationship described above, namely, $1-\left(1-k_{1}t\right)e^{k_{1}t}=t^{2+\beta }$.

## 2. Results

To evaluate the killing kinetics of *E. coli* bacterial populations to ampicillin, we used the following strains: *E. coli* K12 MG1655 Δ*ara* strain, sensitive to all antibiotics, as the experimental control (wt), a spontaneous mutant resistant to nalidixic acid (strain Nal), a spontaneous mutant resistant to rifampicin (strain Rif), a double mutant resistant to both antibiotics (strain NalRif), a strain carrying the plasmid R702 (strain R702) or the plasmid RN3 (strain RN3), as well as *E. coli* strains carrying both chromosomal mutations and the plasmid R702 (strain NalRifR702) or RN3 (strain NalRifRN3). None of these plasmids confer ampicillin resistance.

### 2.1. The Persistence Phase of wt E. coli Starts 230 Minutes After Ampicillin Exposition

**Figure 1:**
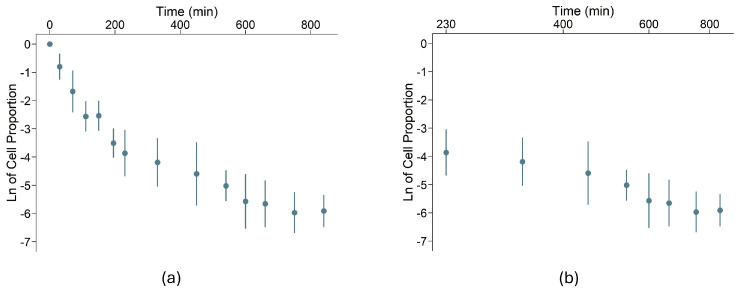
Decay of a wild-type *E. coli* strain population in the presence of ampicillin. In a logarithmic scale, the vertical axis represents the proportion of bacteria still alive (colony-forming units) relative to the initial cell number. Error bars represent the standard deviation. (**a**) The horizontal axis represents time in minutes on a linear scale; (**b**) The horizontal axis represents time in minutes between *t* = 230 min and *t* = 840 min on a logarithmic scale, focusing on the persistence phase.

We analyzed the decay of the wild-type (wt) *E. coli* strain over 840 min (Figure 1). The killing kinetics of the wt strain followed a biphasic pattern [25,26]. Initially, the bacterial population decayed rapidly during the first 230 min in the presence of ampicillin. Then, the persistence phase, where the bacterial population decayed more slowly, was observed from *t* = 230 min until the end of the experiment.

On log-linear scales, the data points from the first phase closely fit a straight line (*r*^2^ = 0.96), indicating that the bacterial population decayed exponentially ($\sim \;e^{k_{1}t}$), with a decay constant of *k₁* = −0.0162 min⁻¹. In the persistence phase, assuming the persister population also decays exponentially ($\sim \;e^{k_{2}t}$), the decay constant was *k₂* = −0.0038 min⁻¹ (*r*^2^ = 0.95). Alternatively, if the persister population decay follows a power law relationship ($\sim \;t^{\beta }$), the decay rate can be estimated by measuring the slope of the data points in log–log scales. In this case, *β* = −1.80 (*r*^2^ = 0.95). When comparing our results with the literature, we observe that the mean value of *β* here obtained (−1.8) does not differ statistically from the value (*β* **=** −2) predicted by Şimşek and Kim in ref. [17] (*t*-test, *p* = 0.31 after a Shapiro–Wilk test, *p* = 0.13), or the value (*β* = −2.1) experimentally obtained by the same authors (*t*-test, *p* = 0.14) [17].

To select which model better explains the persister population decay, we compared the *r*^2^ values of both fittings. Since the calculated *r*^2^ values were similar and close to one (0.96 and 0.95 for exponential and power law fittings, respectively), it was impossible to select a model for the wt strain. Distinguishing whether an exponential or power law distribution better describes persister population decay often requires longer observation periods. To address this, we optimized our protocol to extend the analysis of bacterial decay up to six days, a duration that more closely resembles therapeutic regimens used in clinical settings [27].

### 2.2. Antibiotic-Resistance Determinants Impact Bacterial Death Rates

**Figure 2:**
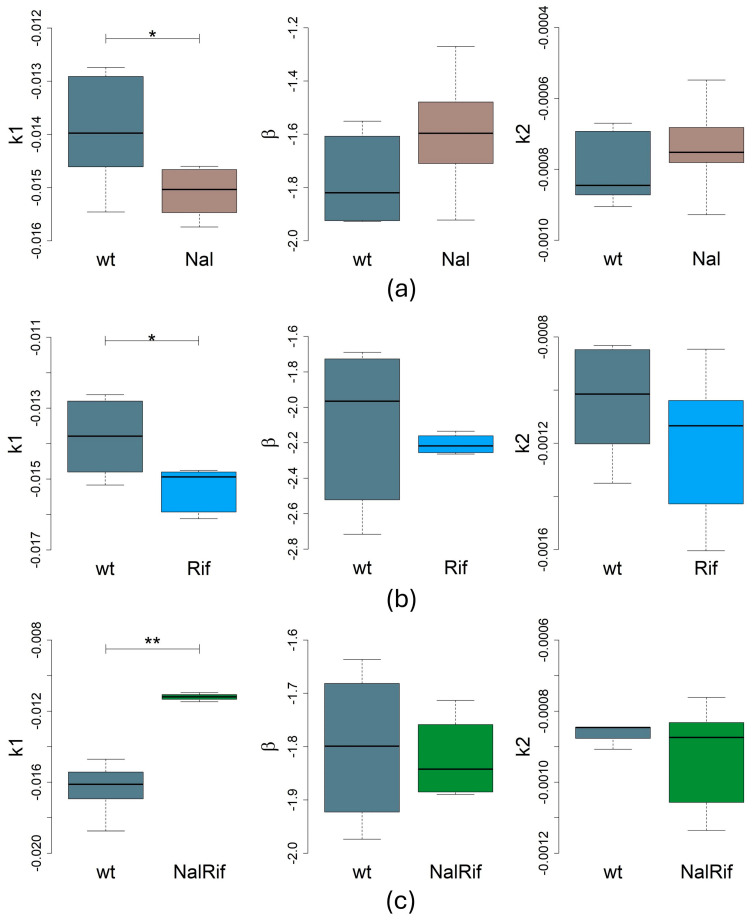
Statistical analysis of decay rates *k*_1_, *k*_2_, and *β* with boxplots of the *E. coli* strains with chromosomal mutations conferring antibiotic resistance. The boxplots show the median and quartiles. We tested normality with Shapiro–Wilk tests of *k*_1_, *β*, and *k*_2_ (Tables S1–S5), and according to its results we used a Student’s *t*-test test with unequal variances or a Wilcoxon test to compare the strains. Significance: *, *p* < 0.05; **, *p* < 0.01. (**a**) Statistical analysis of Nal and wt strains; *n* = six biological replicates for each strain. (**b**) Statistical analysis of Rif and wt strains; *n* = six biological replicates for each strain. (**c**) Statistical analysis of NalRif and wt strains; *n* = five biological replicates for each strain.

**Figure 3:**
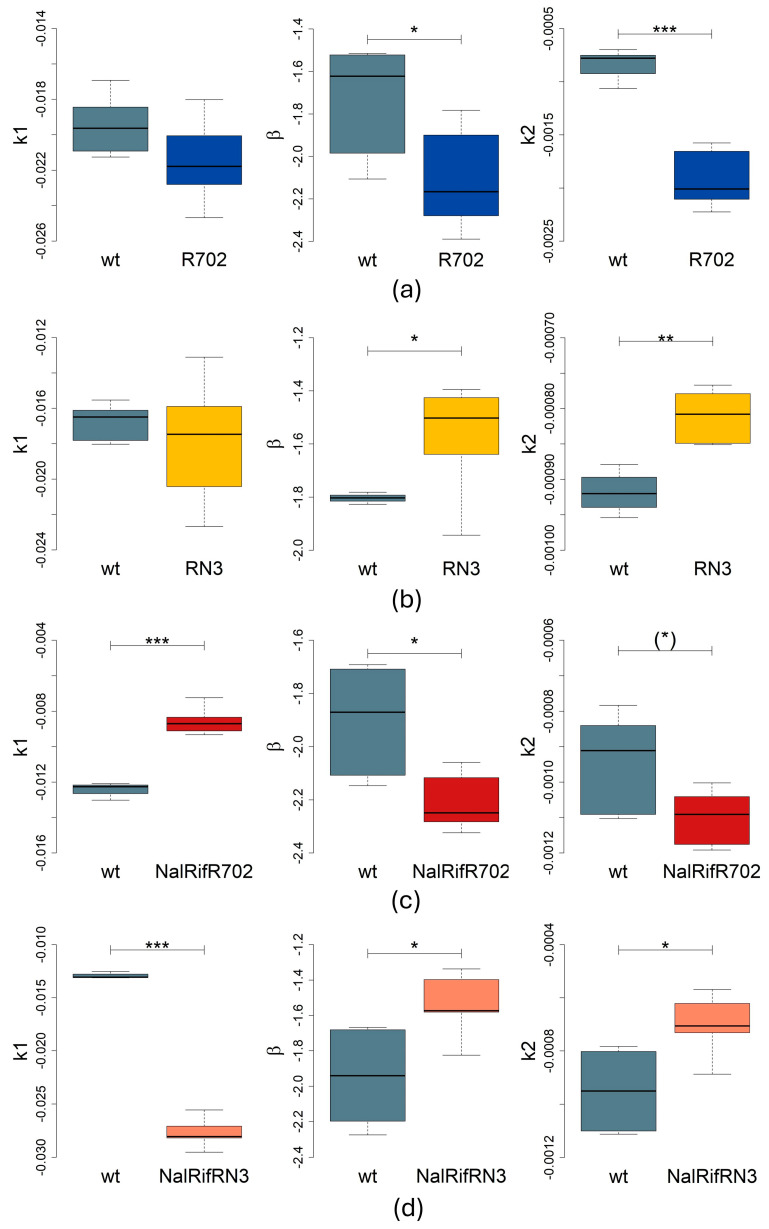
Statistical analysis of decay rates *k*_1_, *k*_2_, and *β* with boxplots of the *E. coli* strains with plasmids conferring antibiotic resistance. The boxplots show the median and quartiles; *n* = six biological replicates for each strain. We tested normality with Shapiro–Wilk tests of *k*_1_, *β*, and *k*_2_ (Tables S1–S5), and according to its results we used a Student’s *t*-test test with unequal variances or a Wilcoxon test to compare the strains. Significance: *, *p* < 0.05; **, *p* < 0.01; ***, *p* < 0.001; (*), *p* = 0.085. (**a**) Statistical analysis of *E. coli* (R702) and wt strains. (**b**) Statistical analysis of *E. coli* (RN3) and wt strains. (**c**) Statistical analysis of NalRifR702 and wt strains. (**d**) Statistical analysis of NalRifRN3 and wt strains.

We then determined the decay constants of the antibiotic-resistant strains and compared them to those of the wild-type strain. Figures S1–S7 show the decay of resistant strains in the presence of ampicillin. We considered that the persistence phase started and goes from *t* = 230 min until the end of the experiment. Table 1 collects all regression values and *p*-values by comparing the decay rate of each strain in each phase with the wt strain. The death rates for the wt and resistant strains without plasmid and with plasmid are depicted in Figure 2 and Figure 3, respectively.

#### 2.2.1. Impact on the First Death Phase

The death rates of R702 or RN3 strains were similar to the wt strain in the first death phase (*t*-tests, *p* > 0.05, Table 1, Figure 3). However, both the Nal and Rif strains (without plasmids) declined faster than the wt strain (*t*-tests, *p* = 0.043 and *p* = 0.018, respectively). The NalRif double mutant declined less than the wt strain (*t*-test, *p* = 0.0019, Table 1, Figure 2). Similarly, the NalRifR702 population declines less than the wt population in this phase (*t*-test, *p* = 9.49 × 10^−5^, Table 1, Figure 3), the opposite of NalRifRN3 populations that decline faster than the wt in the first phase (Wilcoxon test, *p* = 0.0095, Table 1, Figure 3).

#### 2.2.2. Impact on the Second Death Phase (Persister Population)

In the persistence phase, we analyzed data assuming a power law and an exponential. Therefore, there are two *p*-values associated with each case.

In the absence of chromosomal mutations, both plasmids impact the death rate in the persistence phase, but in opposite ways: *E. coli* persistent cells with the R702 plasmid decline faster than wt persister cells (*t*-tests, *p* < 0.05, Table 1, Figure 3), whereas *E. coli* persistent cells with the RN3 plasmid were more resilient than the wt persistent population (*t*-tests, *p* < 0.05, Table 1, Figure 3).

Interestingly, the NalRifR702 strain declined faster in the persistence phase than the wt strain (*t*-tests, *p* = 0.032 assuming a power law, or marginally significant if one assumes an exponential decay in the phase, *p* = 0.085, Table 1, Figure 3), even if these mutations (Nal, Rif, or NalRif) did not affect the decay rate in the persistence phase (*p* > 0.05, Table 1, Figure 2). However, the NalRifRN3 persistent population declined less in the persistence phase than the wt (*t*-tests, *p* < 0.02, Table 1, Figure 3).

**Figure 4:**
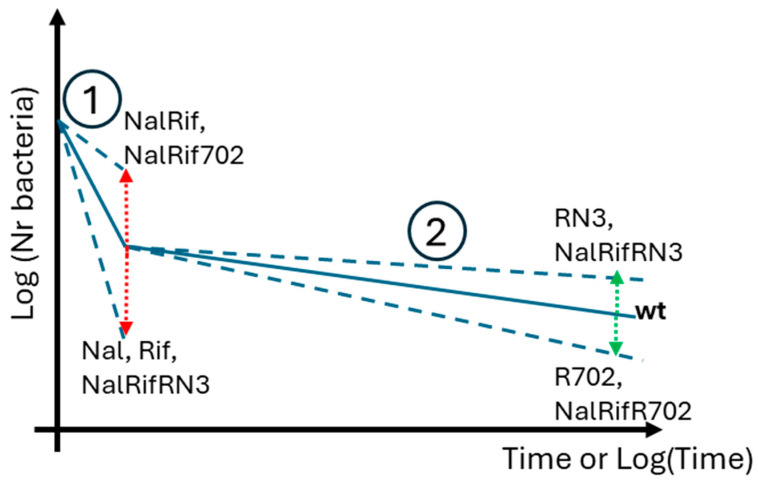
Schematic summary of the different consequences of resistance determinants in *E. coli* death rates. Red arrows indicate statistically significant changes in *k*_1_, while green arrows represent statistically significant or marginally significant changes in *k*_2_, or *β*. Numbers 1 and 2 mark the exponential and persistence phases, respectively. Note that this is a schematic representation of the main results; the values depicted here may not correspond directly to those in Table 1.

Overall, our results show various effects of mutations and plasmids on both death phases. These resistance determinants can affect the first exponential death phase, the death rate of the persistent population, or both phases, as summarized in Figure 4.

### 2.3. Death Rates Do Not Correlate with Growth Rates

**Figure 5:**
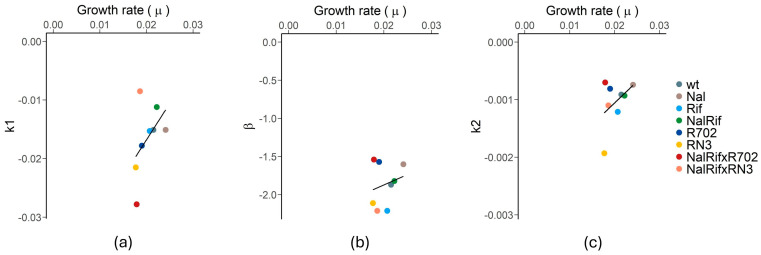
Correlations between growth and death rates. (**a**) Correlation between *µ* and *k*_1_ (*r*^2^ = 0.23, *ρ* = 0.57, and *p* = 0.14). (**b**) Correlation between *µ* and *β* (*r*^2^ = 0.052, *ρ* = 0.29, and *p* = 0.50) (**c**) Correlation between *µ* and *k*_2_ (*r*^2^ = 0.19, *ρ* = 0.096, and *p* = 0.82).

Bacterial cells sensitive to ampicillin are expected to die in the presence of this drug only when replicating. We hypothesized that the death rates observed when cells are exposed to ampicillin would correlate with their growth rate when this drug is absent. To test this hypothesis, we measured the growth rate of each strain and calculated the doubling times in the respective exponential phases (Figure S8). To address if the growth and death rates are intertwined, we performed a correlation analysis between the mean values of the growth rate (*µ*) of each bacterial strain and the mean values of their decay constants (*k*_1_, *β,* and *k*_2_). We performed a Tukey post hoc test after a Shapiro–Wilk test to evaluate their normality (Table S7) and an ANOVA (Table S8). The correlation analysis was performed using the Pearson Correlation (*r*^2^) and the Spearman Rank Correlation (*ρ*). Both Pearson and Spearman approaches revealed very low correlation coefficients and high *p*-values (for *µ-k*_1_: *r*^2^ = 0.23 and *ρ* = 0.57, *p* = 0.14; for *µ-β*: *r*^2^ = 0.052 and *ρ* = 0.29, *p* = 0.50; and for *µ-k*_2_: *r*^2^ = 0.19 and *ρ* = 0.096, *p* = 0.82) (Figure 5). Therefore, no correlation exists between the growth rate (*µ*) and decay constants.

We also measured the correlation between the decay constants, having obtained high correlation rates between *k*_2_ and *β* (*r*^2^ = 0.51, *ρ* = 0.87, and *p* = 0.0045), which was expected since both constants are calculated for the second decay phase (Figure S9). Moreover, we did not find a correlation between *k*_1_ and *k*_2_ (as *r*^2^ = 0.0026 and *ρ* = −0.46) (Figure S9).

### 2.4. The Unexpected Relationship Between k_1_ and β

As persister cells transition in and out of a lag phase via distinct pathways, their rejuvenation kinetics are expected to exhibit significant variability [17]. Mathematically, this verbal argument implies that persister subpopulations should decay proportionally to *t^β^*, where *β* = −2.0, that is, proportionally to $\frac{1}{t^{2}}$ [17]. However, because the heterogeneous subpopulation rejuvenates later than the non-persister cells, their rejuvenation constant should be lower than the latter, *k*_1_ [23]. This means that the death rate in the first phase relates to the death rate in the persistence phase because the population should decay according to $\frac{1-\left(1-k_{1}t\right)e^{k_{1}t}}{t^{2}}$ (note that *k*_1_ < 0 and *t* > 0).

**Figure 6:**
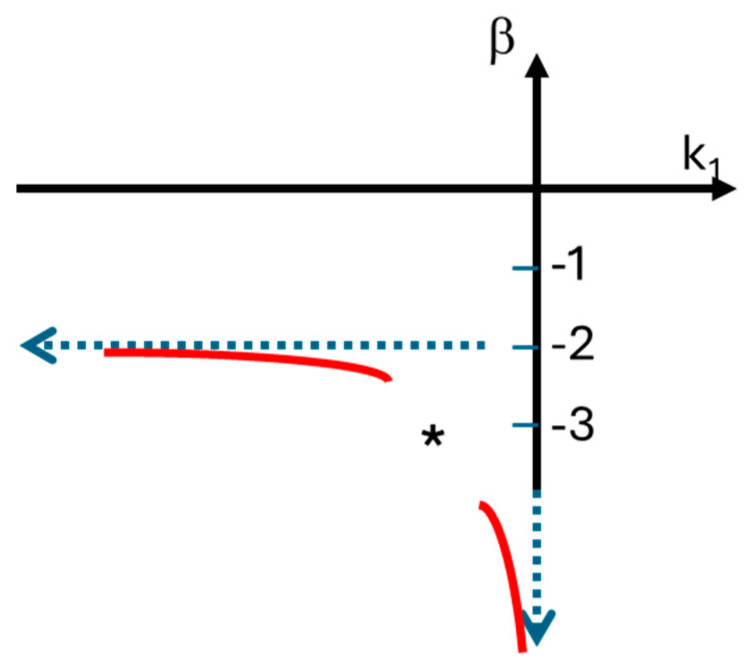
The mathematical relationship between the decay rate of the persister population, *β*, and the initial exponential decay rate, *k*_1_. This function, in red, is always below −2 and is close to −2 for lower values of *k*_1_, the rejuvenation constant of the non-persister population. The monotonic function decreases to −∞ when *k*_1_ goes to zero. For intermediate values of *k*_1_, we do not know how this function behaves (marked with *), but it is still monotonic.

The numerator, $1-\left(1-k_{1}t\right)e^{k_{1}t}=1-\left(1+q\right)e^{-q}$, is an increasing function of −*k*_1_, *t*, and q = −*k*_1_t. Moreover, $0<1-\left(1-k_{1}t\right)e^{k_{1}t}<1$, for all *k*_1_ < 0 and *t* > 0. Therefore, $\frac{1-\left(1-k_{1}t\right)e^{k_{1}t}}{t^{2}}\approx \frac{1}{t^{2+\varepsilon \left(k_{1}\right)}}$, where $\beta =-\left(2+\varepsilon (k_{1}\right))\leq -2$ approaches the value −2 as *k*_1_ becomes more negative values of *k*_1_ (that is, *β* → −2 when *k*_1_ → −∞). Moreover, β decreases when *k*_1_ increases. As such, *β* and *k*_1_ should correlate negatively but not necessarily linearly. Moreover, according to this model *β* ≤ −2 for all *k*_1_ < 0 and *t* > 0. On the other hand, *β* → −∞ when *k*_1_ → 0. Figure 6 shows what we know about this mathematical relationship between *β* and *k*_1_.

**Figure 7:**
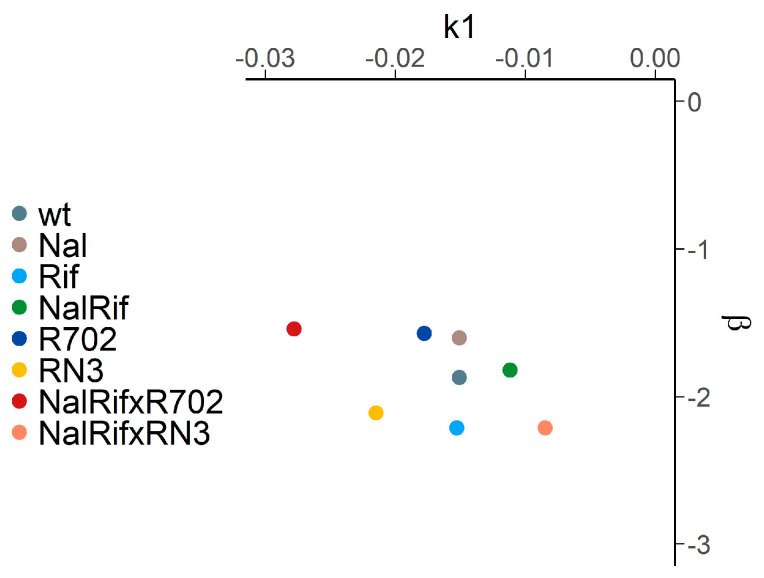
Experimental relationship between the decay rate of the persister population, *β*, and the initial exponential decay rate, *k*_1_.

Moreover, in agreement with the mathematical model, when we analyzed our experimental results, we did not find a linear relationship between *k*_1_ and *β* (Rho = −0.19, Figure 7)).

## 3. Discussion

Most bacteria of clinical interest are resistant to at least one antibiotic [16]. Therefore, it is fundamental to understand the impact of chromosomal resistance mutations and plasmids on bacterial death rates. With this aim, we compared the death rates of strains with and without these resistance determinants following exposure to a bactericidal antibiotic.

Our results show that chromosomal mutations and/or naturally isolated conjugative plasmids conferring antibiotic resistance can affect the decay of a bacterial population exposed to ampicillin, to which they are not resistant. These resistance determinants can affect the first exponential death phase, the death rate of the persistent population, or both phases. For example, both Nal and Rif populations decline faster than the wt populations in the first death phase but not in the persistence phase. Both plasmids impact the death rate in the persistence phase, one increasing (R702) and the other (RN3) decreasing the death rate. As a final example, the NalRifR702 strain declines faster in the first phase but slower in the persistence phase than the wt strain.

We also highlighted evidence of epistatic interactions regarding death rates. For example, while populations of the Nal or Rif strains decline faster than the wt strain, the double mutant NalRif declines slower than the wt strain. Rifampicin resistance is usually caused by mutations in the *rpoB* gene [28,29,30]; however, there are a few mutations known to confer resistance to this antibiotic [29,31]. Therefore, this putative epistatic interaction may be caused by different mutations in the *rpoB* gene in the Rif and NalRif strains. Future work, namely sequencing of the *rpoB* gene in the Rif and NalRif strains, should corroborate or not this assumption. In another case, this time involving the RN3 plasmid, there is more solid evidence of epistatic interactions. The RN3 plasmid has no impact on the death rate of the wt cell in the first death phase, and the double mutant NalRif declines slower than the wt strain; however, the strain NalRifRN3, which has both chromosomal resistances and the RN3 plasmid, declines faster than the wt population. Previous work focusing on bacterial growth rates has shown that epistatic interactions exist between chromosomal mutations conferring resistance to nalidixic acid and rifampicin [31] as well as between these mutations and conjugative plasmids [32] or between plasmids [32,33]. Hitherto, resistance determinants can influence death rates through epistasis, similar to what was previously observed for growing cells.

Ampicillin-sensitive bacterial cells are anticipated to die in the presence of the antibiotic but only during active replication [34,35]. Based on this, we hypothesized that the observed mortality rates of these strains under ampicillin exposure would be linked to their growth rates in the absence of the drug. However, for example, the strains Rif, R702, NalRifR702, or NalRifRN3, which present lower growth rates than the wt strain, decay faster in the first phase than the wt strain. Are these cases outliers or exceptions to a correlation between growth and death rates? To our surprise, growth rates did not correlate with death rates in either the initial death phase or the persistence phase. Like other β-lactam antibiotics, ampicillin can induce the SOS response, a DNA damage repair system, in bacteria. This is primarily due to its interference with cell wall synthesis, which indirectly leads to DNA damage and triggers the SOS pathway [36]. Moreover, the SOS response is activated by DNA-damaging agents and environmental stressors, leading to the formation of persisters that can endure hostile conditions, including antibiotic exposure [37,38]. These relationships between ampicillin, stress, SOS response, and persistence may explain the lack of correlation between death (when facing ampicillin) and growth rates. Another factor possibly implicated in our results is pleiotropy, namely an effect related to the stationary phase and the interaction with the sigma factor RpoS, which has previously been shown to occur precisely with the mutations studied here, namely in the *rpoB* and *gyrA* genes [39,40,41,42,43,44].

We developed a theoretical model and experimentally validated it to test the unexpected relationship between *k*_1_ and *β*, that is, between the death rate in the first phase and the death rate in the second phase (assuming a power law). Importantly, the mathematical formulation makes several new predictions: First, for all *k*_1_ values, *β* should be slightly lower than −2. Experiments reveal that some *β* values may be above −2, although some are not significantly different from the wt (Table 1). The two highest values involve the RN3 plasmid, so these peculiar values may be related to the gene contents of the plasmid; Second, *β* is lower when *k*_1_ is higher and vice versa; this implies that Spearman’s rho should be negative, which is the case (Figure 7); Third, *β* should always be close to −2, which is the case, as all values are between −2.21 and −1.54. These predictions are only partially fulfilled. Moreover, the *ρ*^2^ and *r*^2^ values for both *β* and *k*_2_ were very high (very close to one). Therefore, our results would not be enough to decide whether the persistent population decays according to negative power laws or exponentials.

**Figure 8:**
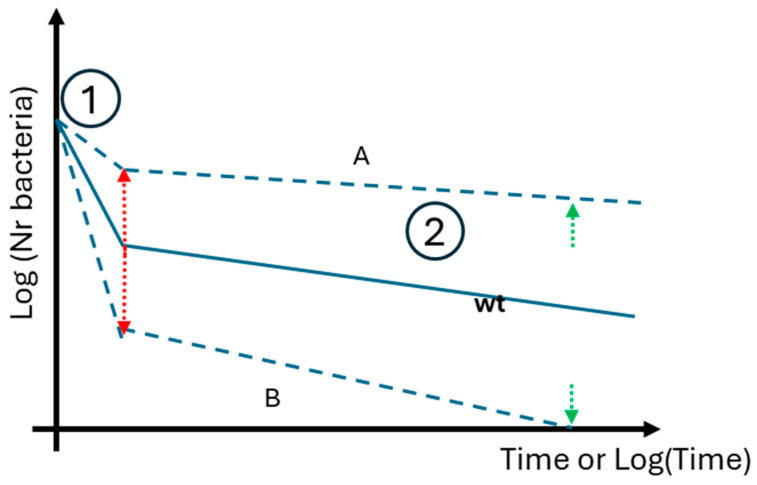
A schematic summary of the “bad” and “good” impacts of resistance determinants. Red arrows indicate changes in *k*_1_, while green arrows represent *k*_2_, or *β*. Numbers 1 and 2 mark the exponential and persistence phases, respectively. (A): This is the bad scenario, where a putative resistance determinant “protects” a bacterial pathogen in both phases 1 and 2 when facing a bactericidal antibiotic to which the pathogen is not resistant. (B): This is a good scenario where a putative resistance determinant disadvantages a bacterial pathogen in both phases (1) and (2) when exposed to a bactericidal antibiotic to which the pathogen is not resistant. In this case B, the antibiotic is significantly more effective at killing cells resistant to other antibiotics than cells sensitive to all antibiotics (wt).

Our findings underscore yet another consequence of antibiotic resistance determinants. They show that antibiotics may exhibit greater efficacy in killing bacteria resistant to other antibiotics than wild-type cells, suggesting their potential as a targeted approach to combat resistant pathogens. Furthermore, the coexistence of multiple resistance determinants within the same cell can lead to epistasis, which may alter the antibiotic’s bactericidal effectiveness. By understanding these dynamics, innovative strategies can be developed to address the challenges posed by antibiotic-resistant bacteria across diverse settings. Figure 8 highlights an important avenue for future research, illustrating two potential scenarios: a ’bad’ scenario and a ’good’ scenario regarding the impact of resistance determinants. In the ’good’ scenario, a putative resistance determinant weakens a bacterial pathogen in both phases of antibiotic-induced death. This means the bactericidal antibiotic is more effective at killing cells resistant to other antibiotics than cells sensitive to all antibiotics. If a conjugative plasmid with this dual advantage can be identified, it could be introduced into pathogens as a prelude to antibiotic treatment, enhancing efficacy. However, in this exploratory study with a small sample size, none of the tested resistance determinants fully aligned with either scenario A or B, underscoring the need for further investigation.

This work highlights the complexity of bacterial resistance and underscores the need for careful consideration of resistance interactions when selecting treatment strategies. The insights gained here could have far-reaching implications for improving bacterial eradication protocols in clinical medicine, enhancing animal health interventions, and optimizing safety measures in industrial food production.

## 4. Materials and Methods

To study the impact of antibiotic resistance conferred by resistance determinants, namely chromosomal mutations and conjugative plasmids, on the death kinetics of bacterial populations and their persistence, we performed time-killing assays. In these assays, we used ampicillin to promote bacterial death. Importantly, none of the tested strains was resistant to this antibiotic.

### 4.1. Bacterial Strains and Plasmids

The *E. coli* K12 MG1655 Δ*ara* was used as the control wt strain and was also used to develop the strains with chromosomal mutations and plasmids.

To develop the *E. coli* strains containing the R702 and RN3 plasmids, the auxotrophic R702 and RN3 plasmid-bearing *E. coli* strains, kindly provided by Prof. Max Mergeay (Belgian Nuclear Research Centre), were used as plasmid donor cells, and *E. coli* K12 MG1655 Δ*ara* as recipient cells. Briefly, the recipient and donor bacteria were grown in LB medium at 37 °C under agitation at 250 rpm for 24 h. Then, 1 mL of each culture was added to 8 mL of LB medium and incubated for 2 h at 37 °C. The conjugation tube was then vortexed, and 100 µL of the cultures (R702 or RN3) were inoculated in plates with Luria agar (LA) supplemented with tetracycline (20 µg/mL). After incubating at 37 °C for 24 h, a colony was re-spread in LA supplemented with the same antibiotics and incubated for 24 h at 37 °C. This process was performed one more time.

The Nal and Rif strains were obtained by plating an overnight *E. coli* K12 MG1655 Δ*ara* culture on LA plates supplemented with nalidixic acid or rifampicin, respectively. Clones of Nal and Rif-resistant mutants were isolated similarly to those described above. The NalRif strain was obtained by plating an overnight culture of the Nal strain on LA plates supplemented with rifampicin. NalRif clones were isolated, as described previously. Glycerol stocks of all strains were prepared and kept at −20 °C. To develop the strains resistant to rifampicin (Rif) and nalidixic acid (Nal), we inoculated the *E. coli* K12 MG1655 Δ*ara* strain in plates with Luria Agar (LA) supplemented with rifampicin (100 µg/mL) or nalidixic acid (40 µg/mL), respectively. After incubating at 37 °C for 24 h, a colony was re-spread in LA supplemented with the same antibiotic and incubated for 24 h at 37 °C. This process was repeated once. Similarly, the double-resistant mutant NalRif was obtained by plating the Nal strain in LA supplemented with rifampicin (100 µg/mL). The respective clone was isolated as described above.

To obtain the strains containing both chromosomal mutations and a plasmid, we performed a conjugation protocol between NalRif (recipient bacteria) and *E. coli* (RN3) or *E. coli* (R702), donor bacteria. The recipient and donor bacteria were grown in LB medium at 37 °C under agitation at 250 rpm for 24 h. Then, 1 mL of each culture was added to 8 mL of LB medium and incubated for 2 h at 37 °C. The conjugation tube was then vortexed, and 100 µL of the cultures (NalRifRN3 or NalRifR702) were inoculated in plates with LA supplemented with rifampicin (100 µg/mL), nalidixic acid (40 µg/mL), and tetracycline (20 µg/mL) to select the transconjugant cells. Clone isolation followed the same steps described previously.

After isolating all the clones, a colony of each strain was inoculated overnight (ON) at 37 °C and 250 rpm in LB medium, and the grown cultures were used to make 30% glycerol stocks, stored at −20 °C until further use.

### 4.2. Bacterial Cell Culture for the Time-Killing Assays

**Figure 9:**
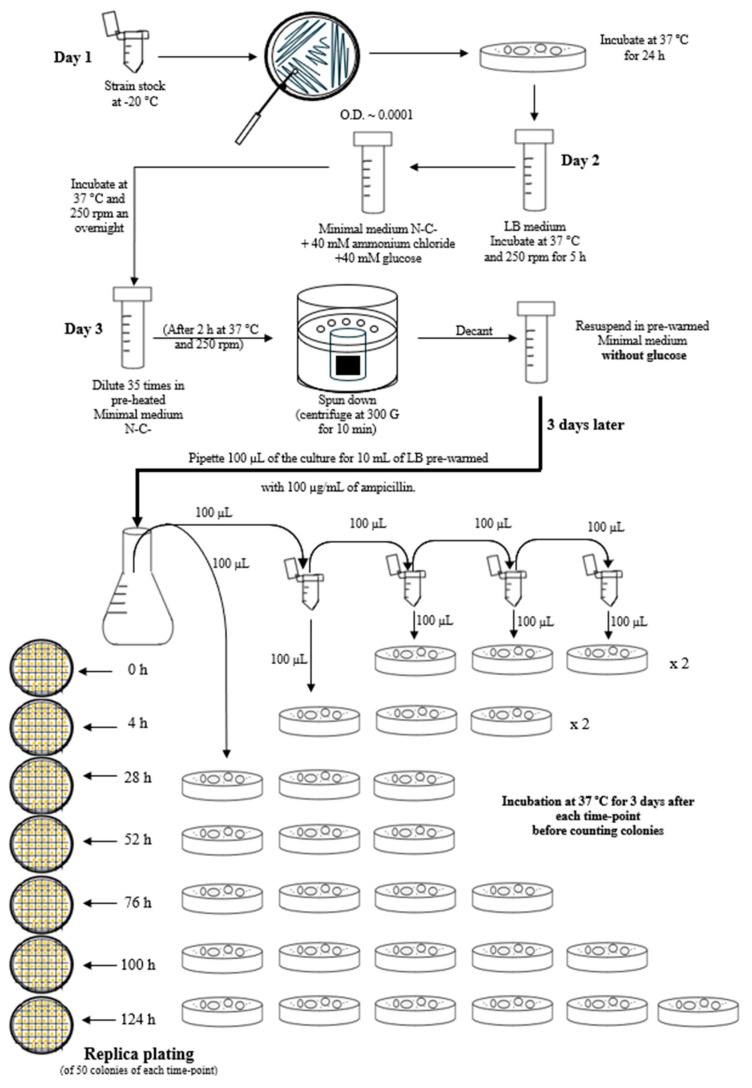
Bacterial cell culture and time-killing assays.

Cells were grown in LB medium at 37 °C and 250 rpm. To monitor their growth, we measure the cultures’ optical density (OD 600 nm) in a Genesys10UVspectrophotometer (ThermoFisher Scientific, Madison, WI, USA). The detailed protocol is illustrated in Figure 9. Briefly, on the first day of cell culture, the bacterial cells were taken from their stocks at −20 °C, spread in plates with LA medium, and incubated for 24 h. The next day, a colony was grown in a falcon with 5 mL of LB medium for 5 h. Next, a small volume (between 1 and 5 µL) of the culture was transferred to a falcon with 5 mL of N^-^C^-^ minimal medium (pH = 7), supplemented with ammonium chloride (40 mM) and glucose (40 mM), at very low densities (such as OD 600 nm ~ 0.0001) and cultured overnight. The low densities ensured that the cultures remained in the exponential phase the following day. The next day, the cultures were diluted at 1:35 and sub-cultured in pre-warmed fresh glucose (40 mM) M9 minimal medium. The cultures were then incubated at 37 °C at 250 rpm for 2 h before they were spun down by centrifugation at 300 G for 10 min. Then, the cell pellet was resuspended in M9 minimal medium without glucose for three days. At the beginning of the third day of starvation, 100 µL of the culture was transferred to a fresh prewarmed LB medium containing ampicillin (100 µg/mL) from Sigma-Aldrich (St. Louis, MO, USA) for the time-killing assays (Figure 9). This concentration was chosen because none of the strains used in this study was able to form colonies on plates with 10 µg/mL of ampicillin.

### 4.3. Time-Killing Assays and Replica Plating

To perform the time-killing assays, 100 µL of the bacterial cultures left in the starvation medium were inoculated in 10 mL of LB supplemented with 100 µg/mL of ampicillin and incubated at 37 °C with a 250 rpm agitation rate for six days. To quantify bacterial cell death, samples were taken at 0 h, 4 h, and every 24 h following ampicillin addition and inoculated in LA plates incubated at 37 °C for 72 h. For each assay, the death kinetics of the wt and the antibiotic-resistant strain were analyzed, and six biological replicates of each strain were performed.

To evaluate if the bacterial cells of the different strains maintained their antibiotic resistance determinants (chromosomal mutations and/or the plasmid), a random biological replicate of each strain was selected in each experiment. After the time-killing assays, fifty colonies of each time point were re-plated in LA medium supplemented with the respective antibiotics for which the strains were resistant (according to the mutations and/or plasmid), and growth was monitored.

### 4.4. Growth Curves

**Figure 10:**
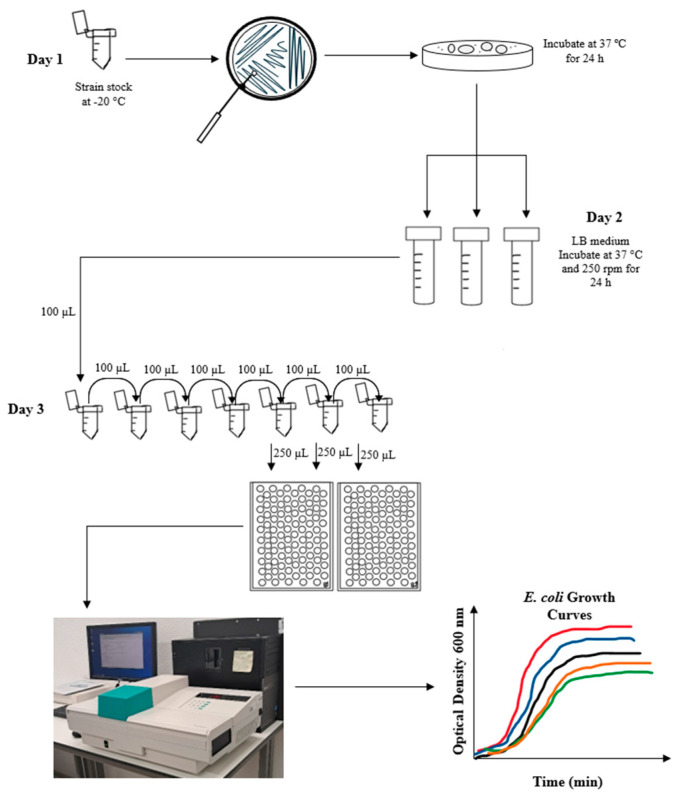
Bacterial growth curves obtained by using Bioscreen. Methodology used to obtain the growth curves.

Growth curves were performed as illustrated in Figure 10. Briefly, three biological replicates of each strain (wt, Nal, Rif, NalRif, R702, RN3, NalRifR702, and NalRifRN3) were incubated ON in LB at 37 °C and 250 rpm (Figure 10). Afterward, serial dilutions of the ON cultures were performed in LB, and 250 µL of each was inoculated in a honeycomb microplate per well. For each biological replicate, three serial dilutions were inoculated. LB was used as a blank control. The microplates were incubated for 24 h at 37 °C with agitation in a microplate reader (Bioscreen). The bacterial growth was assessed by measuring optical density (OD 600 nm) in 10-min intervals. To determine the initial CFUs/mL in each sample, we inoculated the same dilutions in plates with LA medium and counted the colonies after 24 h of incubation at 37 °C.

### 4.5. Mathematical Analysis and Data Processing

Exponential decays are described by the equation N(t) = N_0_.e^k.t^, where the exponential decay constant *k* is negative, N is the number of bacterial cells (CFUs/mL) at a time *t*, and N_0_ = N(*t* = 0) is the initial number of bacterial cells (CFUs/mL) when *t* = 0 min. It is possible to obtain a decreasing straight line with slope *k* by applying the logarithm function on both sides of this equation. The following equation gives a power law decay, N (t) = N_0_.t^β^, where *β* is negative. In this case, one can obtain a straight line with slope *β* by applying the logarithm on both axes (that is, on N(t) and on *t*). Therefore, we obtained the *k*_1_, *β*, and *k*_2_ rates by measuring the slopes of these lines when calculating the logarithm of the dependent variable (to obtain *k*_1_ and *k*_2_) or both variables (to obtain *β*).

We used R-studio v.3.5.1 (available at http://www.rstudio.com/, accessed on 1 April 2024) to analyze the boxplots, remove outliers, and perform statistical analysis.

### 4.6. Statistical Analysis

We used a Shapiro–Wilk test to evaluate the normality of the decay constants of the six biological replicates of both strains studied in an experiment. Also, to compare the decay of the bacterial strains analyzed in each experiment, we used a Student’s two-sample *t*-test for independent samples with unequal variances or a Wilcoxon test, depending on the result of the normality test.

We measured each strain’s growth rate by determining the absorbance values at OD 600 nm (three replicates). A Shapiro–Wilk test was performed to evaluate the normality of the growth rate values, followed by a one-way ANOVA and Tukey’s HSD post hoc test in RStudio (using the tukey_hsd function) to compare the growth rates among the strains.

To analyze the correlations between *µ*, *k*_1_, *β*, and *k*_2_, we performed a Pearson correlation and Spearman rank correlation by using the mean values of each strain for each constant after applying a Shapiro–Wilk test to evaluate the normality of these values.

For all the statistical tests used, we consider α = 0.05.

## 5. Conclusions 

This study demonstrates that chromosomal resistance mutations and conjugative plasmids conferring antibiotic resistance impact bacterial death rates when exposed to ampicillin and, likely, to other antibiotics to which they are not resistant. These resistance determinants affect both the initial exponential death phase and the persistence phase. We also discovered epistatic interactions between these determinants.

Our findings challenge the expected correlation between growth and death rates, suggesting alternative influences like the SOS response and pleiotropy. Although more experimental confirmation is required, theoretical modeling gave us some intuition on patterns of bacterial mortality. Future studies should use these findings to enhance antibiotic efficacy. How? By weakening bacterial pathogens by taking advantage of resistance determinants that are already present.

## Figures and Tables

**Table 1:** Constants describing decay rates of bacterial strains and comparison with the wild-type strain.

Strain	*k*_1_ (min^−1^)	*p* ^1^	*k*_2_ (min^−1^)	*p* ^1^	*r* ^2^	*β*	*p* ^1^	*r* ^2^
Nal	−0.0151	0.043	−0.00074	0.38	0.93	−1.60	0.14	0.96
wt	−0.0139		−0.00081		0.91	−1.77		0.97
Rif	−0.0153	0.018	−0.00121	0.34	0.97	−2.21	0.58	0.88
wt	−0.0138		−0.00104		0.98	−2.10		0.87
NalRif	−0.0112	0.0019	−0.00093	1.00 *	0.93	−1.82	0.83	0.87
wt	−0.0164		−0.00087		0.96	−1.80		0.83
R702	−0.0215	0.11	−0.00193	1.6 × 10^−5^	0.93	−2.11	0.021	0.87
wt	−0.0195		−0.00083		0.97	−1.73		0.92
RN3	−0.0178	0.48	−0.00081	0.0024	0.94	−1.57	0.043	0.72
wt	−0.0167		−0.00092		0.99	−1.80		0.82
NalRifR702	−0.0085	9.5 × 10^−5^	−0.00110	0.085	0.98	−2.21	0.032	0.87
wt	−0.0124		−0.00095		0.91	−1.91		0.81
NalRifRN3	−0.0278	0.0095 *	−0.00070	0.012	0.91	−1.54	0.014	0.96
wt	−0.0129		−0.00095		0.95	−1.95		0.89

^1^ The *p*-values marked with * were calculated with the non-paramedic Wilcoxon test. The remaining *p*-values were calculated using the parametric Student’s *t*-test.

## Data Availability

All data are in the paper or in the Supplementary Material.

## Supplementary Materials

The following supporting information can be downloaded at www.mdpi.com/article/10.3390/antibiotics14020201/s1: Figure S1: Killing curves of wt and Nal strains; Figure S2: Killing curves of wt and Rif strains; Figure S3: Killing curves of wt and NalRif strains; Figure S4: Killing curves of wt and R702 strains; Figure S5: Killing curves of wt and RN3 strains; Figure S6: Killing curves of wt and NalRifR702 strains; Figure S7: Killing curves of wt and NalRifRN3 strains; Table S1: Shapiro–Wilk Test for values of the logarithm of cell proportions of wt groups use for plots of *k*_2_; Table S2. Shapiro–Wilk Test for values of the logarithm of cell proportions of the other *E. coli* strains groups use for plots of *k*_2_; Table S3. Shapiro–Wilk Test for values of the logarithm of cell proportions of wt groups use for plots of *β*. Table S4. Shapiro–Wilk Test for values of the logarithm of cell proportions of the other *E. coli* strains groups use for plots of *k*_2_. Table S5. Shapiro–Wilk Test for the values of the constants of all replicates. Figure S8: Results of the analysis of growth curves; Table S6. Results of the Tukey test for the growth rates (*µ*). Table S7. Shapiro–Wilk Test for the growth rates (*µ*) of the different strains studied. Table S8. One-way ANOVA of growth rate (*µ*) values of the different bacteria. Table S9. Results of Shapiro–Wilk Test for all the parameters analyzed. Figure S9: Correlation between the decay constants.
