# Supplementary material for: What Is the Impact of Antibiotic Resistance Determinants on the Bacterial Death Rate?
Source: Antibiotics (Basel). 2025 Feb 14;14(2):201. doi: 10.3390/antibiotics14020201 (PMC11851504; doi:10.3390/antibiotics14020201)
Supplement: Supplementary file 1 [file antibiotics-14-00201-s001.zip › antibiotics-3426571-supplementary.pdf]

Supplementary Materials for the *Article*:

What is the impact of antibiotic resistance determinants on the bacterial death rate?

Bruno S. Luz [1], João S. Rebelo [1] , Francisca Monteiro [1,*] and Francisco Dionisio [1,*]

[1] cE3c—Centre for Ecology, Evolution and Environmental Changes & CHANGE, Global Change and Sustainability Institute, Faculdade de Ciências, Universidade de Lisboa,
1749-016 Lisboa, Portugal; bruno luz255@gmail.com (B.S.L.); joaorebelo_4@hotmail.com (J.S.R.); fsmonteiro@fc.ul.pt (F.M.)

* Correspondence: fsmonteiro@fc.ul.pt and fadionisio@fc.ul.pt

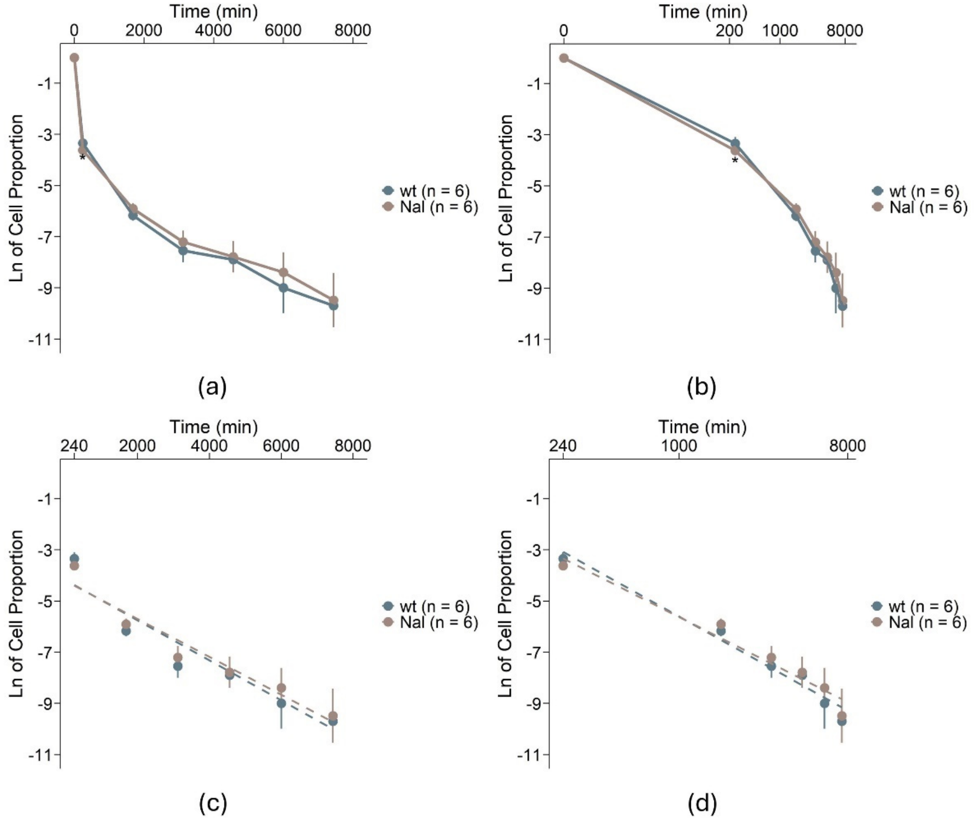

Figure S1. Killing Curves of wt and Nal strains. Killing curves of the strains wt (in grey) and Nal (in brown) on log or linear scales and statistical analysis of decay constants k_1, k_2, and β; n = 6 biological replicates for each strain. We tested normality with Shapiro-Wilk tests of k_1, β, and k_2, and proportions at each time-point (Table S1-S5). The error bars correspond to the standard deviation. (**a**) All data on a log-linear scale; asterisks mark statistical differences with the Student's T-test test with unequal variances; (t = 4 h): p-value = 0.044. (**b**) All data on a log-log scale; asterisks as in (a). (**c**) The second phase on a log-linear scale, hence assuming exponential decay; dashed line represents the linear regressions for k_2 for wt (y = -0.00081x – 4.1813, R^2 = 0.91) and Nal (y = -0.00074x – 4.2198, R^2 = 0.91). (**d**) The second phase on a log-log scale, hence assuming decay as a power law; dashed line represents the linear regressions for β for wt (y = -1.77x + 6.6266, R^2 = 0.91) and Nal (y = -1.60x + 5.4324, R^2 = 0.96).

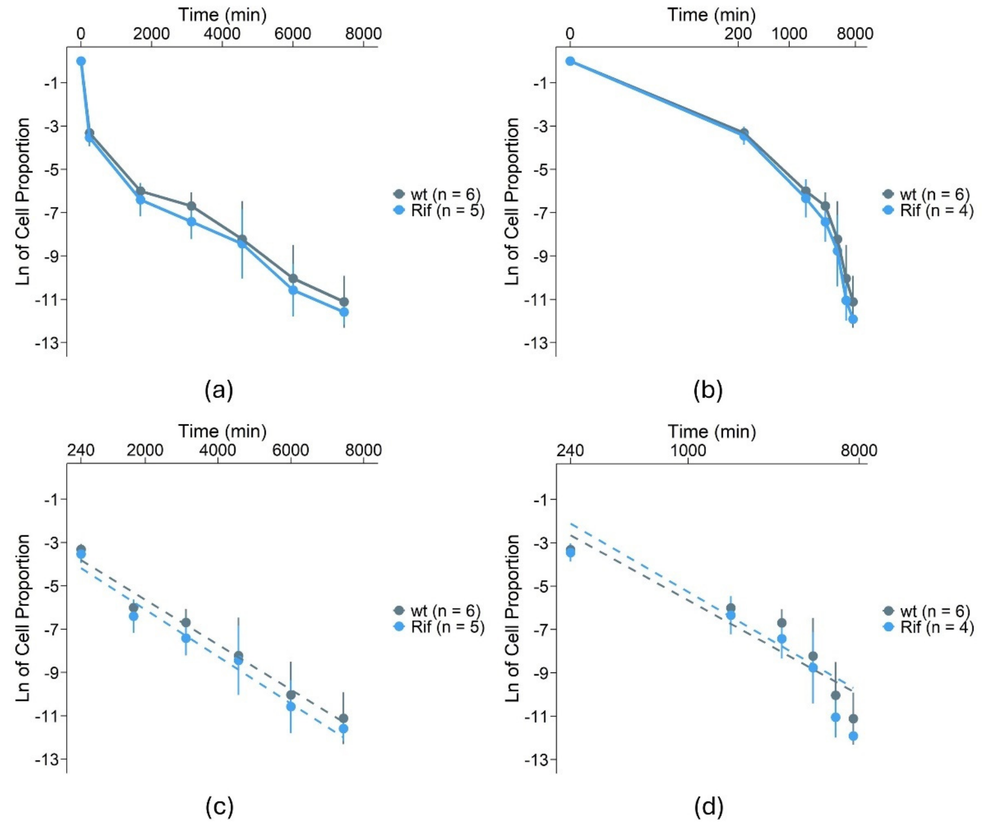

Figure S2. Killing Curves of wt and Rif strains. Killing curves of the strains wt (in grey) and Rif (in blue) on log or linear scales and statistical analysis of decay constants k_1, k_2, and β; n = 6 biological replicates for wt and n = 5 or n= 4 for Rif. We tested normality with Shapiro-Wilk tests of k_1, β, and k_2, and proportions at each time-point (Table S1-S5). The error bars correspond to the standard deviation. (**a**) All data on a log-linear scale, and no statistical differences were found. (**b**) All data on a log-log scale; and no statistical differences were found. (**c**) The second phase on a log-linear scale, hence assuming exponential decay; dashed line represents the linear regressions for k_2 for wt (y = -0.00104x – 3.5591, R^2 = 0.98) and Rif (y = -0.00121x – 3.8939, R^2 = 0.97). (**d**) The second phase on a log-log scale, hence assuming decay as a power law; dashed line represents the linear regressions for β for wt (y = -2.10x + 8.8602, R^2 = 0.87) and Rif (y = -2.21x + 10.003, R^2 = 0.88).

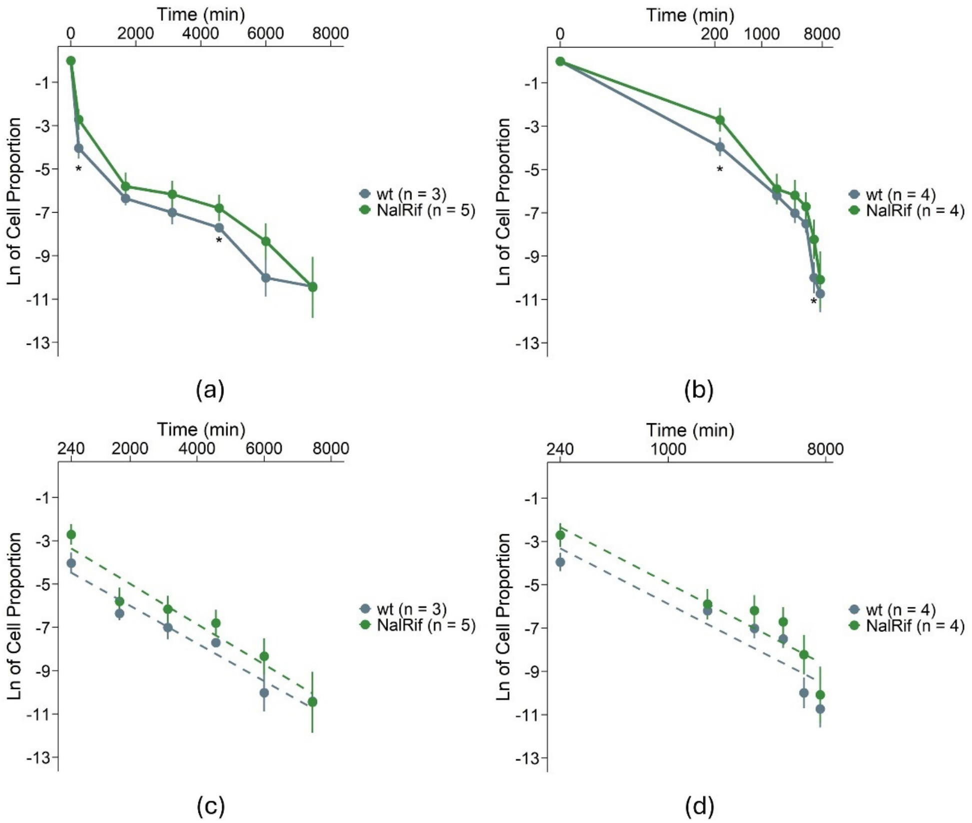

Figure S3. Killing Curves of wt and NalRif strains. Killing curves of the strains wt (in grey) and NalRif (in green) on log or linear scales and statistical analysis of decay constants k_1, k_2, and β; n = 3 or n = 4 biological replicates for wt and n = 5 or n= 4 biological replicates for Rif. We tested normality with Shapiro-Wilk tests of k_1, β, and k_2, and proportions at each time-point (Table S1-S5). The error bars correspond to the standard deviation. (**a**) All data on a log-linear scale; asterisks mark statistical differences with the Student's T-test test with unequal variances; from left to right (t = 4 h, 76 h): p-value = 0.018; p-value = 0.028. (**b**) All data on a log-log scale; the asterisks from left to right (t = 4 h, 100 h): p-value = 0.013; p-value = 0.024. (**c**) The second phase on a log-linear scale, hence assuming exponential decay; dashed line represents the linear regressions for k_2 for wt ($y = -0.00087x - 4.2656$, $R^2 = 0.96$) and NalRif ($y = -0.00093x - 3.1353$, $R^2 = 0.93$). (**d**) The second phase on a log-log scale, hence assuming decay as a power law; dashed line represents the linear regressions for β for wt ($y = -1.80x + 6.5461$, $R^2 = 0.83$) and NalRif ($y = -1.82x + 7.6331$, $R^2 = 0.87$).

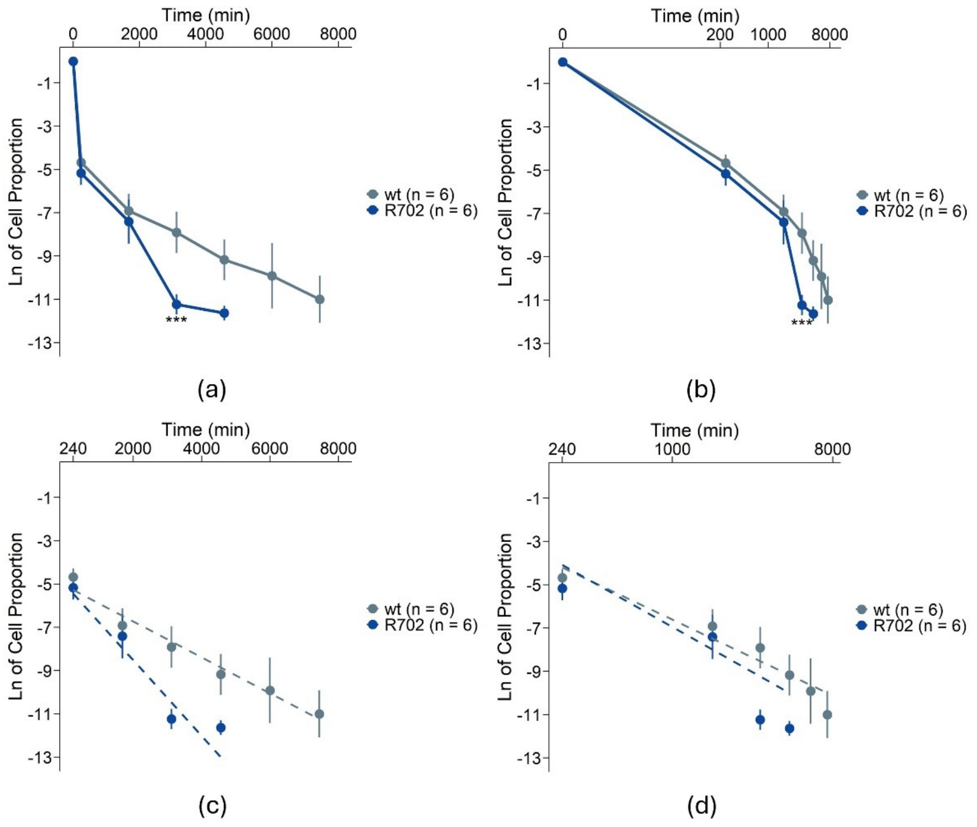

Figure S4. Killing Curves of wt and R702. Killing curves of the strains wt (in grey) and R702 (in blue) on log or linear scales and statistical analysis of decay constants k_1, k_2, and β; n = 6 biological replicates for each strain. We tested normality with Shapiro-Wilk tests of k_1, β, and k_2, and proportions at each time-point (Table S1-S5). The error bars correspond to the standard deviation. We failed to obtain colonies of *E. coli* (R702) when t = 100 h and t= 124 h. (**a**) All data on a log-linear scale, asterisks mark statistical differences with the Student's T-test test with unequal variances; (t = 52 h): p-value = 0.00010. (**b**) All data on a log-log scale; asterisks as in A. (**c**) The second phase on a log-linear scale, hence assuming exponential decay; dashed line represents the linear regressions for k_2 for wt (y = -0.00083x – 5.0708, R^2 = 0.97) and R702 (y = -0.00193x – 4.9866, R^2 = 0.93). (**d**) The second phase on a log-log scale, hence assuming decay as a power law; dashed line represents the linear regressions for β for wt (y = -1.73x + 5.2759, R^2 = 0.92) and R702 (y = -2.11x + 7.4867, R^2 = 0.87).

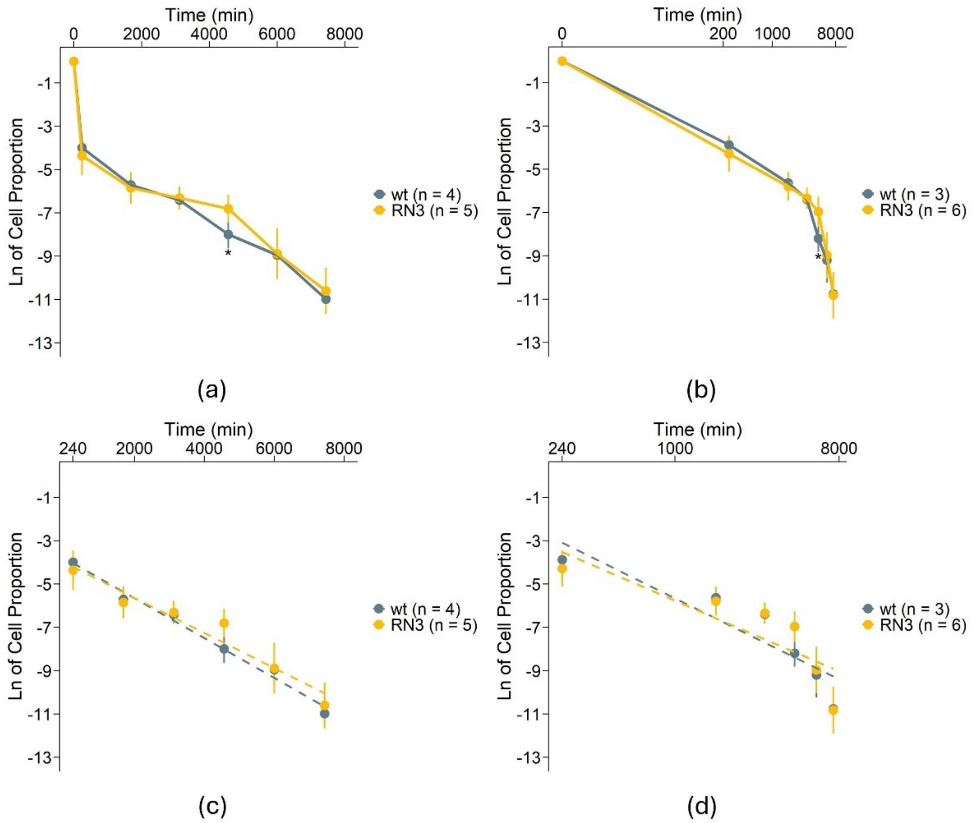

Figure S5. Killing curves of wt and RN3. Killing curves of the strains wt (in grey) and RN3 (in yellow) on log or linear scales and statistical analysis of decay constants k_1, k_2, and β; n = 4 or n = 3 biological replicates for wt and n = 5 or n= 6 biological replicates for RN3. We tested normality with Shapiro-Wilk tests of k_1, β, and k_2, and proportions at each time-point (Table S1-S5). The error bars correspond to the standard deviation. (**a**) All data on a log-linear scale, asterisks mark statistical differences with the Student's T-test test with unequal variances; (t = 76 h): p-value = 0.032. (**b**) All data on a log-log scale; asterisks (t = 76 h): p-value = 0.050. (**c**) The second phase on a log-linear scale, hence assuming exponential decay; dashed line represents the linear regressions for k_2 for wt ($y = -0.00092x - 3.815$, $R^2 = 0.99$) and RN3 ($y = -0.00081x - 4.0211$, $R^2 = 0.94$). (**d**) The second phase on a log-log scale, hence assuming decay as a power law; dashed line represents the linear regressions for β for wt ($y = -1.80x + 6.7819$, $R^2 = 0.82$) and RN3 ($y = -1.57x + 5.0829$, $R^2 = 0.72$).

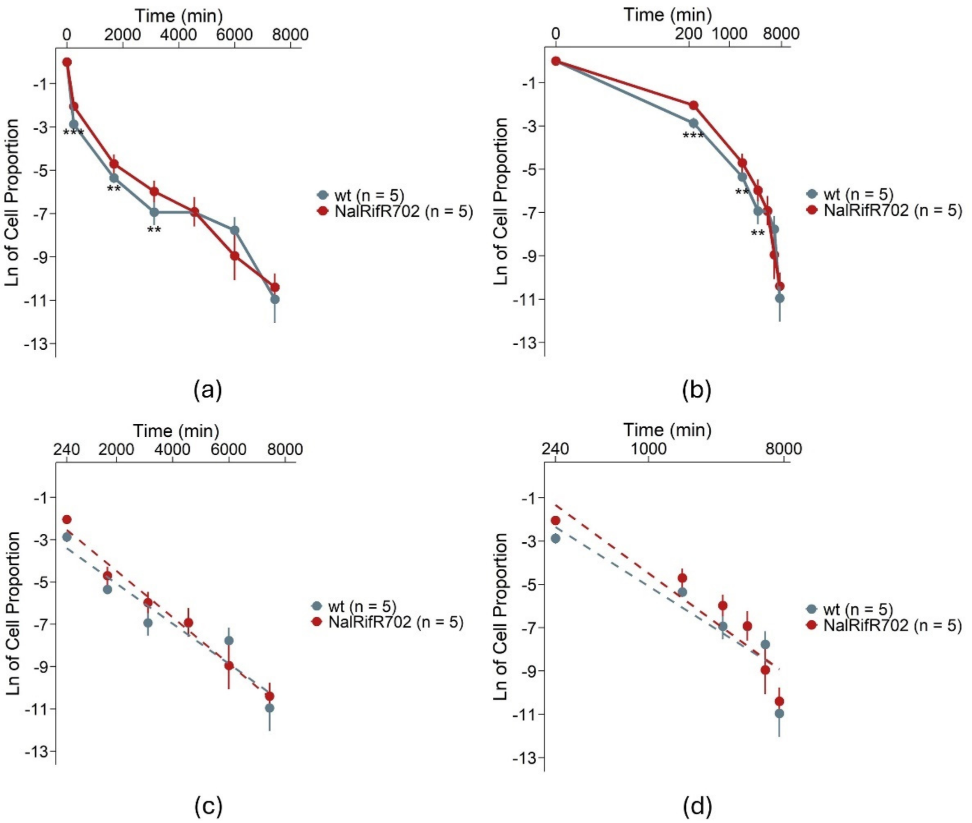

Figure S6. Killing Curves of wt and NalRifR702. Killing curves of the strains wt (in grey) and NalRifR702 (in red) on log or linear scales and statistical analysis of decay constants k_1, k_2, and β; n = 5 biological replicates for each strain. We tested normality with Shapiro-Wilk tests of k_1, β, and k_2, and proportions at each time-point (Table S1-S5). The error bars correspond to the standard deviation. (**a**) All data on a log-linear scale; asterisks mark statistical differences with the Student's T-test test with unequal variances; from left to right (t = 4 h, 28 h and 52 h): p-value = 0.00046; p-value = 0.024 and p-value = 0.027. (**b**) All data on a log-log scale; asterisks as in (a). (**c**) The second phase on a log-linear scale, hence assuming exponential decay; dashed line represents the linear regressions for k_2 for wt ($y = -0.00095x - 3.174$, $R^2 = 0.91$) and NalRifR702 ($y = -0.00110x - 2.2764$, $R^2 = 0.98$). (**d**) The second phase on a log-log scale, hence assuming decay as a power law; dashed line represents the linear regressions for β for wt ($y = -1.91x + 8.1147$, $R^2 = 0.81$) and NalRifR702 ($y = -2.21x + 10.778$, $R^2 = 0.87$).

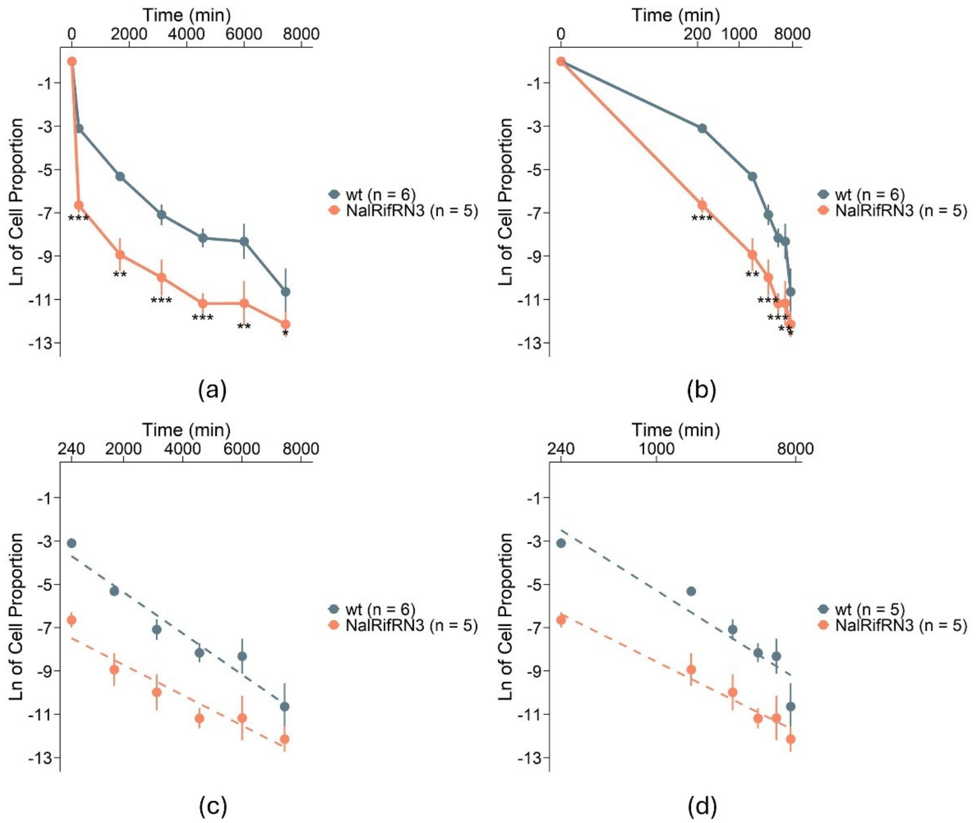

Figure S7. Killing Curves of wt and NalRifRN3. Killing curves of the strains wt (in grey) and NalRifRN3 (in orange) on log or linear scales and statistical analysis of decay constants k_1, k_2, and β; n = 6 biological replicates for wt and n = 5 biological replicates for NalRifRN3. We tested normality with Shapiro-Wilk tests of k_1, β, and k_2, and proportions at each time-point (Table S1-S5). The error bars correspond to the standard deviation. (**a**) All data on a log-linear scale; asterisks mark statistical differences with the Student's T-test test with unequal variances; from left to right (t = 4 h, 52 h, 76 h, 100 h and 124 h): p-value = 4.30×10^{-7}; p-value = 0.00041; p-value = 2.80×10^{-6}; p-value = 0.0015; p-value = 0.019. For t = 28 h, a Wilcoxon-test was applied: p-value = 0.0043. (**b**) All data on a log-log scale; asterisks as in (a). (**c**) The second phase on a log-linear scale, hence assuming exponential decay; dashed line represents the linear regressions for k2 for wt ($y = -0.00095x - 3.4604$, $R^2 = 0.95$) and NalRifRN3 ($y = -0.00070x - 7.3126$, $R^2 = 0.91$). (**d**) The second phase on a log-log scale, hence assuming decay as a power law; dashed line represents the linear regressions for β for wt ($y = -1.95x + 8.1947$, $R^2 = 0.89$) and NalRifRN3 ($y = -1.54x + 2.0712$, $R^2 = 0.96$).

Table S1. Shapiro-Wilk Test for values of the logarithm of cell proportions of wt groups use for plots of k_2.

Time	Statistic	wt (NalRifR702)	wt (NalRifRN3)	wt (NalRif)	wt (Nal)	wt (Rif)	wt (R702)	wt (RN3)
4 h	W	0.84	0.93	1.00	0.95	0.92	0.96	0.98
	p	0.16	0.55	0.88	0.74	0.54	0.79	0.88
28 h	W	0.97	0.90	0.98	0.97	0.99	0.88	0.89
	p	0.86	0.35	0.70	0.88	0.98	0.26	0.39
52 h	W	0.88	0.84	0.89	0.93	0.91	0.84	0.87
	p	0.33	0.14	0.37	0.61	0.42	0.13	0.30
76 h	W	0.94	0.92	0.81	0.94	0.83	0.93	0.87
	p	0.69	0.53	0.14	0.70	0.11	0.55	0.29
100 h	W	0.92	0.92	0.79	0.89	0.88	0.88	0.84
	p	0.54	0.56	0.10	0.31	0.29	0.26	0.21
124 h	W	0.87	0.89	0.82	0.90	0.85	0.80	0.80
	p	0.25	0.29	0.15	0.35	0.15	0.06	0.10

[1] Used to test the normality of the logarithm of cell proportions of the replicates used for plotting the second phase of decay after analysis of k_2 values.

Table S2. Shapiro-Wilk Test for values of the logarithm of cell proportions of the other *E. coli* strains groups use for plots of k_2.

Time	Statistic	NalRifR702	NalRifRN3	NalRif	Nal	Rif	R702	RN3
4 h	W	0.91	0.98	0.93	0.94	0.83	0.99	0.98
	p	0.49	0.91	0.59	0.66	0.14	0.98	0.96
28 h	W	0.99	0.72	0.99	0.89	0.98	0.87	0.94
	p	0.96	0.02	0.96	0.34	0.95	0.24	0.66
52 h	W	0.91	0.86	0.95	0.86	0.95	0.82	0.88
	p	0.45	0.22	0.71	0.18	0.71	0.08	0.31
76 h	W	0.98	0.90	0.87	0.92	0.84	(sample size = 2)	0.90
	p	0.96	0.40	0.26	0.50	0.17		0.39
100 h	W	0.90	0.89	0.91	0.88	0.99	(sample size = 0)	0.94
	p	0.42	0.36	0.46	0.26	0.97		0.63
124 h	W	0.87	0.93	0.93	0.98	0.92	(sample size = 0)	0.88
	p	0.25	0.63	0.57	0.93	0.46		0.33

[1] Used to test the normality of the logarithm of cell proportions of the replicates used for plotting the second phase of decay after analysis of k_2 values.

Table S3. Shapiro-Wilk Test for values of the logarithm of cell proportions of wt groups use for plots of β.

Time	Statistic	wt (NalRifR702)	wt (NalRifRN3)	wt (NalRif)	wt (Nal)	wt (Rif)	wt (R702)	wt (RN3)
4 h	W	0.84	0.93	0.96	0.95	0.92	0.96	0.99
	p	0.16	0.55	0.75	0.74	0.54	0.79	0.84
28 h	W	0.97	0.90	0.97	0.97	0.99	0.88	1.00
	p	0.86	0.35	0.82	0.88	0.98	0.26	0.91
52 h	W	0.88	0.84	0.95	0.93	0.91	0.84	0.76
	p	0.33	0.14	0.69	0.61	0.42	0.13	0.03
76 h	W	0.94	0.92	0.80	0.94	0.83	0.93	0.91
	p	0.69	0.53	0.10	0.70	0.11	0.55	0.41
100 h	W	0.92	0.92	0.88	0.89	0.88	0.88	0.86
	p	0.54	0.56	0.36	0.31	0.29	0.26	0.27
124 h	W	0.87	0.89	0.87	0.90	0.85	0.80	0.78
	p	0.25	0.29	0.30	0.35	0.15	0.06	0.07

[1] Used to test the normality of the logarithm of cell proportions of the replicates used for plotting the second phase of decay after analysis of β values.

Table S4. Shapiro-Wilk Test for values of the logarithm of cell proportions of the other *E. coli* strains groups use for plots of k_2.

Time	Statistic	NalRifR702	NalRifRN3	NalRif	Nal	Rif	R702	RN3
4 h	W	0.91	0.98	0.95	0.94	0.87	0.99	0.98
	p	0.49	0.91	0.73	0.66	0.29	0.98	0.96
28 h	W	0.99	0.72	0.95	0.89	0.96	0.87	0.96
	p	0.96	0.02	0.71	0.34	0.81	0.24	0.81
52 h	W	0.91	0.86	0.96	0.86	0.89	0.82	0.88
	p	0.45	0.22	0.78	0.18	0.37	0.08	0.28
76 h	W	0.98	0.90	0.75	0.92	0.87	(sample size = 2)	0.86
	p	0.96	0.40	0.04	0.50	0.31		0.20
100 h	W	0.90	0.89	0.91	0.88	0.93	(sample size = 0)	0.93
	p	0.42	0.36	0.47	0.26	0.49		0.62
124 h	W	0.87	0.93	0.98	0.98	(sample size = 2)	(sample size = 0)	0.91
	p	0.25	0.63	0.87	0.93			0.43

[1] Used to test the normality of the logarithm of cell proportions of the replicates used for plotting the second phase of decay after analysis of β values.

Table S5. Shapiro-Wilk Test for the values of the constants of all replicates.

Experiment	Statistics	k_1	β	k_2
wt	W	0.80	0.86	0.88
	p	0.11	0.23	0.32
NalRifR702	W	0.91	0.91	0.92
	p	0.49	0.48	0.54
wt	W	0.74	0.91	0.89
	p	0.03	0.43	0.34
NalRifRN3	W	0.94	0.93	0.95
	p	0.62	0.62	0.77
wt	W	0.96	0.97	0.76
	p	0.81	0.85	0.03
NalRif	W	1.00	0.89	0.92
	p	0.91	0.39	0.55
wt	W	0.95	0.87	0.85
	p	0.75	0.23	0.16
Nal	W	0.94	0.99	0.97
	p	0.65	1.00	0.89
wt	W	0.92	0.85	0.86
	p	0.54	0.15	0.20
Rif	W	0.80	0.92	0.96
	p	0.07	0.56	0.84
wt	W	0.96	0.82	0.88
	p	0.79	0.09	0.29
R702	W	0.99	0.95	0.91
	p	0.98	0.72	0.43
wt	W	0.92	1.00	0.98
	p	0.52	0.95	0.93
RN3	W	0.98	0.85	0.88
	p	0.96	0.15	0.31

[1] Used to test the normality of the values of the decay constants of all replicates considered after removal of outliers.

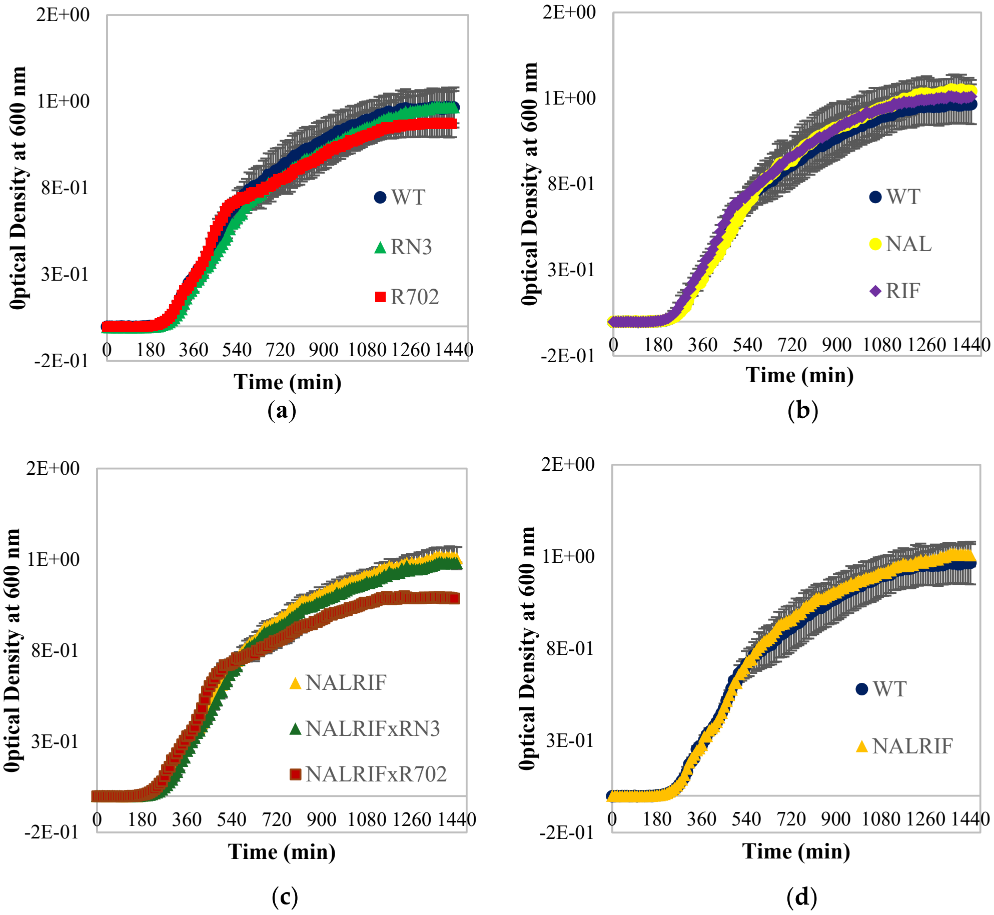

2E+00
1E+00
8E-01
3E-01
-2E-01
Optical Density at 600 nm
0 180 360 540 720 900 1080 1260 1440
Time (min)
WT
RN3
R702
(a)
WT
NAL
RIF
(b)
NALRIF
NALRIFxRN3
NALRIFxR702
(c)
WT
NALRIF
(d)

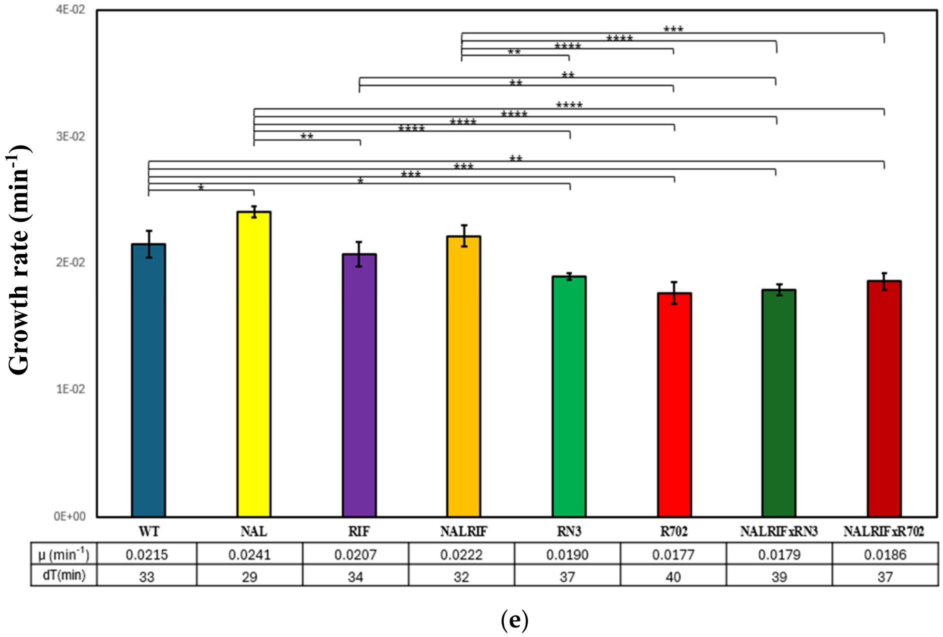

	WT	NAL	RIF	NALRIF	RN3	R702	NALRIFxRN3	NALRIFxR702
μ (min^{-1})	0.0215	0.0241	0.0207	0.0222	0.0190	0.0177	0.0179	0.0186
dT(min)	33	29	34	32	37	40	39	37

(**e**)

Figure S8. Results of the analysis of growth curves. (**a**), (**b**), (**c**) and (**d**) show the growth curves of each *E. coli* K12 MG1655 Δara strain studied after diluting 10^{-5} the original cultures. Each curve represents the mean of the three biological replicates for the absorbance at an OD 600 nm in time-points separated by 10 min from the instant t = 0 min to t = 24 h. (**e**) shows the average of growth rates of the different bacteria and their doubling time, and the results of a Tukey-Test used to compare these values after a Shapiro-Wilk Test (Table S7), and an one-way ANOVA (Table S8).

Table S6. Results of Tukey-Test for the growth rates (μ).

Strain 1	Strain 2	p-value	Asterisks
Nal	NalRif	1.07e-01	ns
Nal	NalRifR702	3.75e-06	****
Nal	NalRifRN3	7.36e-07	****
Nal	R702	4.09e-07	****
Nal	Rif	1.32e-03	**
Nal	RN3	9.31e-06	****
Nal	wt	1.43e-02	*
NalRif	NalRifR702	6.51e-04	***
NalRif	NalRifRN3	8.56e-05	****
NalRif	R702	4.11e-05	****
NalRif	Rif	3.51e-01	ns
NalRif	RN3	1.98e-03	**
NalRif	wt	9.55e-01	ns
NalRifR702	NalRifRN3	9.42e-01	ns
NalRifR702	R702	7.71e-01	ns
NalRifR702	Rif	5.40e-02	ns
NalRifR702	RN3	9.99e-01	ns

NalRifR702	wt	5.04e-03	**
NalRifRN3	R702	1.00e+00	ns
NalRifRN3	Rif	6.20e-03	**
NalRifRN3	RN3	6.81e-01	ns
NalRifRN3	wt	5.89e-04	***
R702	Rif	2.70e-03	**
R702	RN3	4.33e-01	ns
R702	wt	2.68e-04	***
Rif	RN3	1.54e-01	ns
Rif	wt	9.11e-01	ns
RN3	wt	1.58e-02	*

Table S7. Shapiro-Wilk Test for the growth rates (μ) of the different strains studied.

Strains	W	*p*
**wt**	0.94	0.54
**Nal**	1.00	0.88
**Rif**	0.96	0.60
**NalRif**	0.92	0.46
**RN3**	0.87	0.30
**R702**	0.88	0.32
**NalRifRN3**	0.96	0.64
**NalRifR702**	0.94	0.54

Table S8. One-way ANOVA of growth rate (μ) values of the different bacteria.

Anova: one-way

SUMARRY

Groups	*Conting*	*Sum*	*Average*	*Variance*
**wt**	3	0.064	0.0215	1.13E-06
**Nal**	3	0.072	0.0241	1.85E-07
**Rif**	3	0.062	0.0207	9.29E-07
**NalRif**	3	0.066	0.0222	7.53E-07
**RN3**	3	0.057	0.0190	8.81E-08
**R702**	3	0.053	0.0177	8.067E-07
**NalRifRN3**	3	0.054	0.0179	1.95E-07

**NalRifR702**	3	0.056	0.0186	4.67E-07

ANOVA

Source of variation	*SQ*	*gl*	*MQ*	*F*	*p-value*	*critical-F*
Between groups	0.00011	7	1.57E-05	27.57	8.79E-08	2.66
Inside groups	9.11E-06	16	5.69E-07			
Total	0.00012	23				

[1] Used to compare the growth rates of the different *E. coli* strains by using three biological replicates.

Table S9. Results of Shapiro-Wilk Test for all the parameters analysed.

**Parameters**	**W**	***p***
**μ**	0.93	0.50
$\mathbf{k_1}$	0.94	0.65
**β**	0.80	0.031
$\mathbf{k_2}$	0.87	0.15

[1] Used to study the normality of the averages of the different bacteria for all the parameter studied.

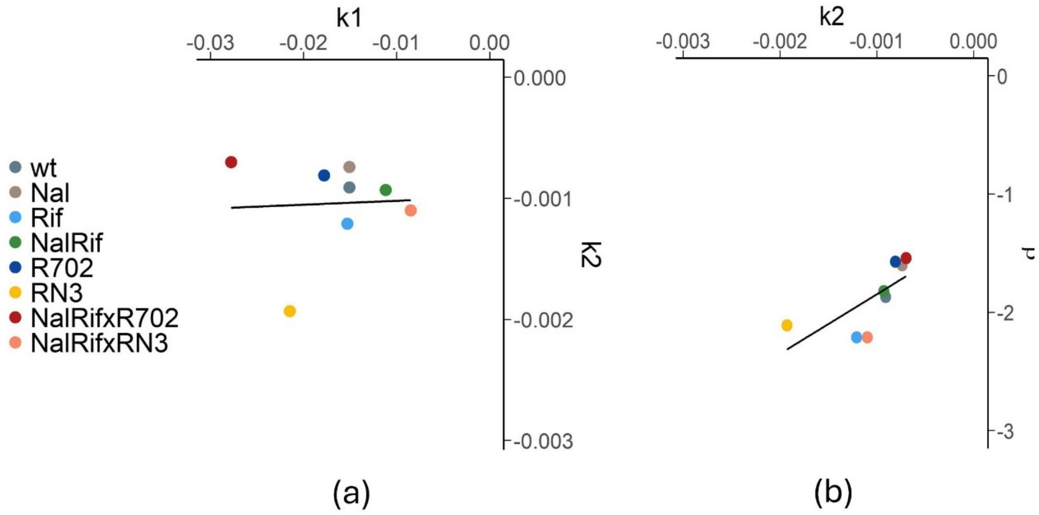

Figure S9. Correlation between the decay constants. After calculating both coefficients of Pearson and Spearman. (**a**) represents the correlation between k_1-k_2: as $R^2 = 0.0026$ and Rho = - 0.46 (p-value = 0.25). (**b**) represents the correlation between k_2-β: as $R^2 = 0.51$ and Rho = 0.87 (p-value = 0.0045).
